# Onset of Quiescence Following p53 Mediated Down-Regulation of H2AX in Normal Cells

**DOI:** 10.1371/journal.pone.0023432

**Published:** 2011-08-12

**Authors:** Yuko Atsumi, Hiroaki Fujimori, Hirokazu Fukuda, Aki Inase, Keitaro Shinohe, Yoshiko Yoshioka, Mima Shikanai, Yosuke Ichijima, Junya Unno, Shuki Mizutani, Naoto Tsuchiya, Yoshitaka Hippo, Hitoshi Nakagama, Mitsuko Masutani, Hirobumi Teraoka, Ken-ichi Yoshioka

**Affiliations:** 1 Division of Genome Stability Research, National Cancer Center Research Institute, Tokyo, Japan; 2 Division of Cancer Development System, National Cancer Center Research Institute, Tokyo, Japan; 3 Department of Pathological Biochemistry, Medical Research Institute, Tokyo Medical and Dental University, Tokyo, Japan; 4 Department of Pediatrics and Developmental Biology, Graduate School of Medical and Dental Sciences, Tokyo Medical and Dental University, Tokyo, Japan; Texas A&M University, United States of America

## Abstract

Normal cells, both *in vivo* and *in vitro*, become quiescent after serial cell proliferation. During this process, cells can develop immortality with genomic instability, although the mechanisms by which this is regulated are unclear. Here, we show that a growth-arrested cellular status is produced by the down-regulation of histone H2AX in normal cells. Normal mouse embryonic fibroblast cells preserve an H2AX diminished quiescent status through p53 regulation and stable-diploidy maintenance. However, such quiescence is abrogated under continuous growth stimulation, inducing DNA replication stress. Because DNA replication stress-associated lesions are cryptogenic and capable of mediating chromosome-bridge formation and cytokinesis failure, this results in tetraploidization. Arf/p53 module-mutation is induced during tetraploidization with the resulting H2AX recovery and immortality acquisition. Thus, although cellular homeostasis is preserved under quiescence with stable diploidy, tetraploidization induced under growth stimulation disrupts the homeostasis and triggers immortality acquisition.

## Introduction

Cancer is a disease associated with genomic instability and the accumulation of mutations [1]. Unlike specific chromosomal translocation-associated tumors, most cancers associated with aging develop either chromosomal instability (CIN) or microsatellite instability (MIN) [2]. While MIN is associated with mismatch repair deficiency, CIN develops even in a normal background [3]. However, the mechanisms by which CIN and MIN develop remain elusive.

A recent genomic analysis of various cancers revealed that massive genomic rearrangements, including loss of heterozygosity (LOH) and chromosomal translocation, amplification and deletion, do not gradually accumulate over time, as conventionally thought, but appear to be acquired in a single catastrophic event [4]. One of such events could be associated with tetraploidization because tetraploidy is a common early event in cancer cells with CIN [5]. Tetraploidy is observed in cells during the initial stages of cancer [6], [7] as well as in precancerous stages such as dysplasia [8], [9], but not in malignant cancer cells, which usually exhibit aneuploidy in association with deploidization [5]. Furthermore, analogous to changes observed in cancer genomes, the immortalization of mouse embryonic fibroblasts (MEFs) occurs with tetraploidy and mutation of the Arf/p53 module, which eventually evolves into aneuploidy during serial cultivation [10].

In the initial stages of carcinogenesis, cells are subjected to oncogenic stress, resulting in the accumulation of DNA replication stress-associated lesions and the onset of barrier responses such as senescence and apoptosis [11], [12]. This effect can be reproduced *in vitro* by the activation of oncogenes [11] and accelerated growth stimulation [12] due to the induction of accelerated S-phase entry and the resulting DNA replication stress. Importantly, genomic instability is generated under these conditions [11], [12] because DNA replication stress-associated lesions persist into M phase and mediate chromosomal bridge formation and cytokinesis failure, resulting in tetraploidization [10]. In fact, tetraploidization of MEFs is induced via chromosomal bridge formation prior to the onset of immortality with mutation of Arf/p53 [10], although it is still unclear how tetraploidization induces immortality. Since such tetraploidization is specifically observed during senescence, tetraploidization might be a defect that occurs during cell proliferation or growth arrest. In fact, similar to cells in the initial stages of carcinogenesis, senescent cells often accumulate irreparable DNA lesions [13], [14] and frequently exhibit genomic instability [15].

The development of cancer, as well as the onset of immortality in cells *in vitro*, is tightly associated with mutations in the Arf/p53 module [16]–[18]. Although this is ascribed to the role of p53 in cancer prevention, the regulation and roles of p53 are complex [18]. While constitutively active p53 mediates premature aging in mice [19]–[21], additional single gene copies of *Arf* and *p53* under functional regulation mediate longevity and cancer prevention [22]. Similarly, while the accumulation of p53 induces cellular senescence and apoptosis [16], [17], additional single gene copies of *Arf* and *p53* in MEFs has a protective effect from immortalization [22], suggesting that they help to maintain homeostasis under undamaged conditions. This raises the questions of the identity of the regulatory target of p53 in preserving cellular homeostasis under normal conditions and how cellular homeostasis preservation and abrogation are associated with genomic status and p53 regulation.

This study focused on the mechanism by which normal cells under serial proliferation regulate homeostasis preservation and abrogation and sought to identify the regulatory target of p53. Our results illustrated two distinct conditions that could result in growth-arrested cells: (i) cells that maintain continuous quiescence by down-regulating H2AX (a variant of core histone H2A) under p53 regulation and stable-diploidy maintenance; and (ii) cells that develop tetraploidy and immortality under continuous growth stimulation, characterized by the accumulation of γH2AX foci. Thus, oncogenic stress under growth stimulation triggers catastrophic tetraploidization that leads to immortalization in association with the accompanying mutation of the Arf/p53 module and recovery of H2AX expression and growth activity.

## Results

### Immortality is prevented in quiescent cells that maintain genomic stability

**Figure 1:**
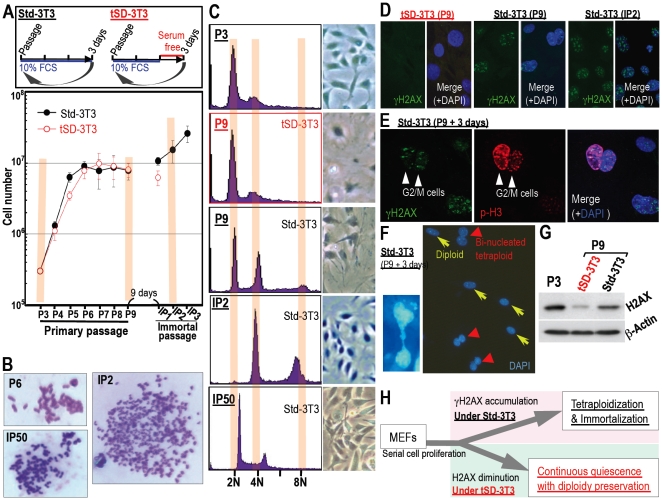
Immortality with tetraploidy is blocked in quiescent cells with diploidy, diminished H2AX, and no γH2AX foci. **A.** Growth curves of MEFs cultured under the standard 3T3 protocol (Std-3T3) or the T3 protocol with temporary serum deprivation (tSD-3T3) as schematically shown. MEFs under Std-3T3 conditions were immortalized, whereas MEFs cultured under tSD-3T3 conditions were not. **B** Genomic instability developed in immortalized MEFs (IP2) under Std-3T3 conditions. **C.** Genomic status was determined by flow-cytometry at the indicated conditions and passages. Representative images are shown. Tetraploidy development was blocked under tSD-3T3 conditions, while tetraploidy had already developed in growth-arrested MEFs at P9 under Std-3T3 conditions (see increasing 4N and 8N peaks). **D.** DNA lesions identified by γH2AX foci spontaneously accumulated in MEFs developing tetraploidy and immortality (P9) under Std-3T3 conditions as well as in immortal cells (IP2), while MEFs that maintained quiescent status with genomic stability under tSD-3T3 conditions contained no foci. **E.** DNA lesion-carryover into the G2-M phases was determined for lesions that spontaneously accumulated in senescent MEFs under Std-3T3 conditions. DNA lesions in senescing MEFs are also observed in the G2-M phases determined by phosphorylated H3. **F.** Chromosome bridge formation (Left panel) is observed in association with DNA lesion-carryover into the G2-M phases under Std-3T3 conditions with the resulting accumulation of bi-nucleated tetraploidy (Right panel: red arrow heads). Representative images are shown. **G.** The total H2AX level at P9 under each condition was determined. Whereas a significant reduction in H2AX expression was observed in MEFs with genomic stability under tSD-3T3 conditions, MEFs that developed immortality and genomic instability under Std-3T3 conditions did not show a significant decrease in H2AX expression. **H.** A model of the life-cycle of MEFs undergoing quiescence or developing immortality. While quiescent MEFs preserve diploidy and show diminished H2AX levels, MEFs developing immortality exhibited γH2AX foci accumulation.

MEFs cultured under the standard 3T3 protocol (Std-3T3) senesce in association with oxygen sensitivity [23], which is followed by the development of immortality with tetraploidy [10] and mutation of the Arf/p53 module [22], similar to the process of carcinogenesis. In addition, similar to cells in the initial stages of carcinogenesis, spontaneous DNA lesions accumulate in senescent MEFs under Std-3T3 conditions prior to the development of immortality [10], which suggests that growth stimulation induced under Std-3T3 conditions might overwhelm senescent MEFs. Therefore, MEFs under Std-3T3 conditions were compared with MEFs exposed to temporary serum deprivation (tSD-3T3), which induces occasional growth arrest (Fig. 1A). Under Std-3T3 conditions, MEFs were immortalized with tetraploidy that progresses to aneuploidy (Fig. 1A–C). On the other hand, MEFs cultured under tSD-3T3 conditions never developed immortality and preserved quiescence with stable diploidy (Fig. 1A, C). This indicates that temporal growth arrest prevents immortalization and supports genomic stability. Conversely, continuous culture with 10% FBS produces oncogenic stress in senescent MEFs, triggering tetraploidization. Thus, even though both are growth arrested (at least in total cell numbers) with senescent morphology at the same culture passage (P9) (Fig. S1), MEFs under tSD-3T3 conditions are continuously quiescent with genomic stability, while MEFs under Std-3T3 conditions develop tetraploidy (Fig. 1A, C), posing a question in DNA lesion status that induces chromosomal bridge formation and tetraploidization [10].

### γH2AX foci accumulate in cells developing genomic instability but not in cells preserving diploidy

To determine the DNA lesion status induced by accelerated growth stimulation, γH2AX foci were compared in growth-arrested MEFs (P9) under both conditions (Fig. 1D). As expected, MEFs that developed tetraploidy under Std-3T3 conditions accumulated γH2AX foci, with some carrying over into the G2/M phases (Fig. 1E). This resulted in chromosome bridge formation (Fig. 1F) with the resulting tetraploidization that is initially observed with binucleated tetraploidy (Fig. 1F). On the other hand, quiescent MEFs that preserved genomic stability under tSD-3T3 conditions did not develop γH2AX foci (Fig. 1D), indicating that genomic stability is preserved under no γH2AX signal. However, it was still unclear why quiescent MEFs under tSD-3T3 conditions do not accumulate γH2AX foci because senescent cells are known to generally accumulate irreparable DNA lesions [13], [14].

To address why γH2AX foci do not form under tSD-3T3 conditions, the expression level of H2AX at P9 was determined. As shown in Figure 1G, a remarkable reduction in H2AX expression was observed in quiescent MEFs at P9 while MEFs that developed tetraploidy under Std-3T3 conditions showed significantly higher H2AX expression than quiescent MEFs. This illustrates an association between H2AX levels and the cellular and genomic status, in that cells with largely diminished H2AX expression preserve stable diploidy and a quiescent status, while cells with residual H2AX expression and with γH2AX foci develop genomic instability and immortality (Fig. 1H). Importantly, H2AX-KO cells exhibited impaired DNA repair, growth retardation, and elevated genomic instability [24]–[28], phenotypes reminiscent of senescent cells. Therefore, it will be critical to determine how H2AX-status is regulated to produce quiescence and induce genomic instability.

### H2AX is generally diminished in quiescent cells

**Figure 2:**
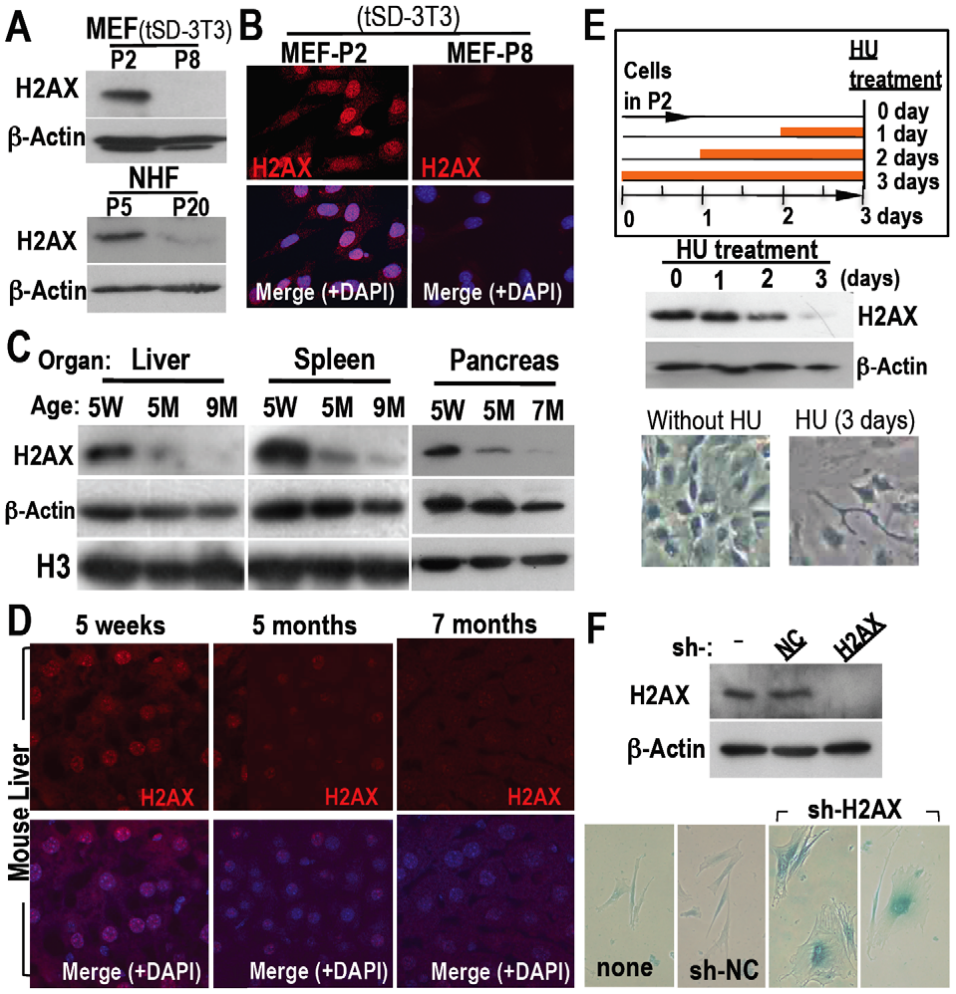
Quiescent cell-status is induced with H2AX diminution both *in vitro* and *in vivo*. **A,B** H2AX expression in growth-arrested cells (P8 for MEFs under tSD-3T3 conditions, P20 for NHFs) was determined by Western blotting (**A**) and immunofluorescent staining (**B**), revealing H2AX diminution in both types of growth-arrested cells. **C,D** H2AX diminution was also measured in adult mice organs by Western blotting (**C**) and in liver sections by immunofluorescent staining (**D**). Samples were prepared from five week (5W), five month (5M) and seven- or nine-month-old mice (7M or 9M). **E.** The involvement of H2AX diminution in DNA damage-induced premature senescence was determined after 0.2 mM HU treatment. Orange bars indicate the periods of HU treatment. Premature damage-induced senescence was observed with H2AX diminution, in which cells were flattened and enlarged, morphology typical of senescent cells. **F.** The effect of H2AX knockdown on senescence was determined in NHFs. Senescence was directly induced by H2AX knockdown in NHFs. H2AX status and senescence was determined by Western blotting (top) and SA-β-gal activation, and cells exhibited a flattened and enlarged morphology (bottoms), respectively.

To address whether H2AX diminution is a general occurrence, H2AX expression was compared in normal human fibroblasts (NHFs) and MEFs. Decreased H2AX was observed in both cell types at growth-arrested stage after serial proliferation (Fig. 2A, B), suggesting that this process is conserved between humans and mice. In addition, H2AX diminution was also observed in many organs of adult mice, including the liver, spleen, and pancreas (Fig. 2C, D; Fig. S2). Thus, H2AX is generally reduced in quiescent cell chromosomes both *in vitro* and *in vivo*.

H2AX is also diminished during premature senescence induced by DNA damage. Using early passage MEFs (P2), H2AX diminution was observed when senescence was induced by treatment with hydroxyurea (HU) to induce DNA replication stress (Fig. 2E) and with the radiomimetic DNA-damaging agent, neocarzinostatin (Fig. S3). This most likely occurs because DNA repair is coupled with H2AX release and chromatin remodeling [29]–[31]. Together with results showing a decrease in H2AX transcript levels in senescent MEFs (Fig. S4), these results indicate that decreased amounts of H2AX protein in senescing cells is ascribed to a decrease in H2AX transcript levels and DNA damage.

To directly address the impact of H2AX reduction, H2AX was knocked down in early passage NHFs, which induced cellular quiescence with senescent cell characteristics; cells adopted a flattened and enlarged morphology and showed an increase in senescence-associated β-galactosidase activity (Fig. 2F). Since the knockdown of H2AX in 293T cells induced growth arrest without inducing a senescent morphology (data not shown), it is likely that the effect of H2AX diminution is primarily due to quiescence induction and potentially a normal consequence of senescence in normal cells.

### Immortalized cells develop following tetraploidization when H2AX status and growth activity are restored

**Figure 3:**
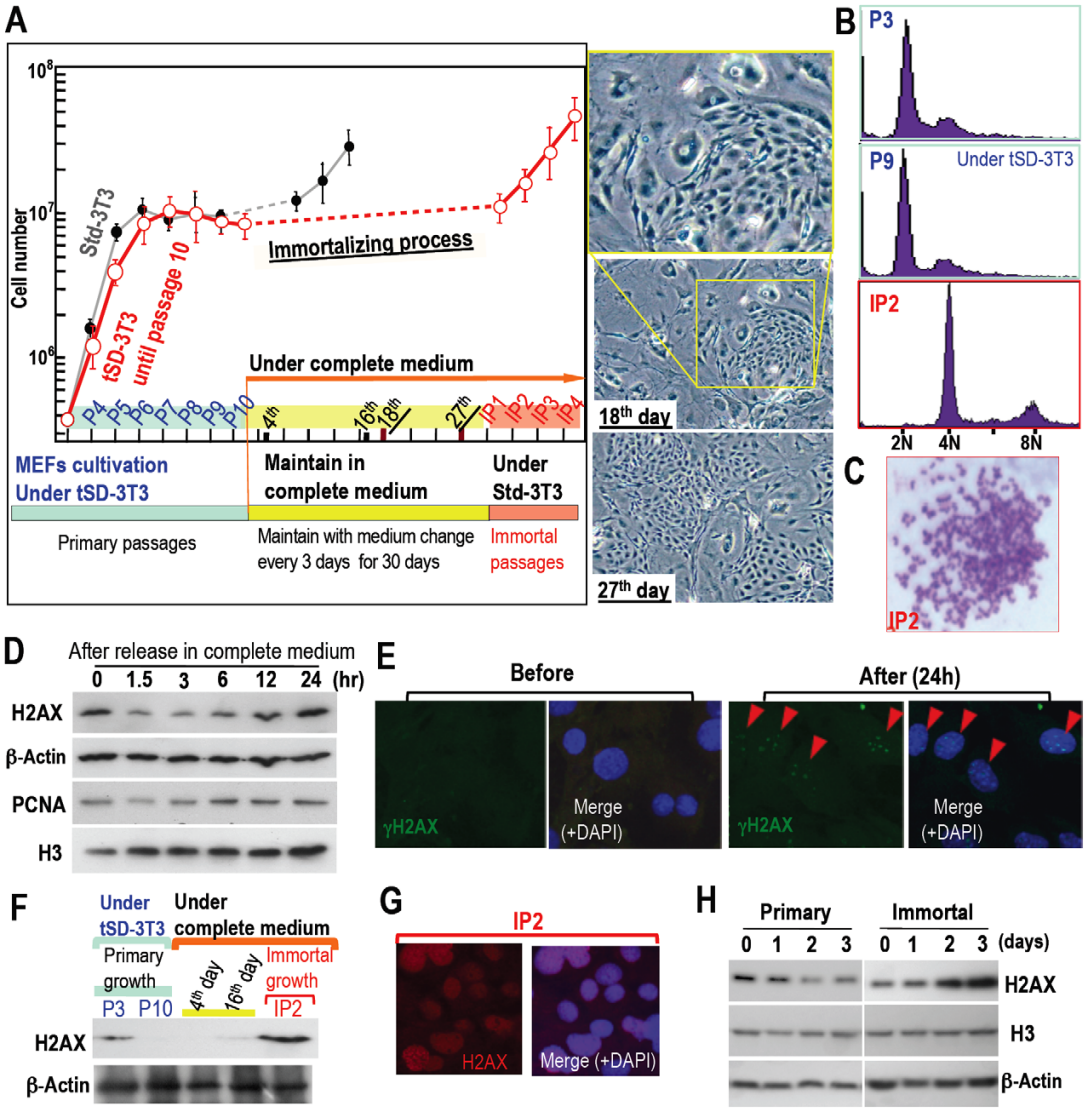
H2AX-diminished quiescent cell-status is abolished by continuous growth stimulation with accompanying H2AX recovery. **A.** Quiescent MEFs with diminished H2AX expression were cultured under tSD-3T3 conditions until P10. They were then exposed to complete medium, which was changed every three days for 30 days. Immortal passages were started under Std-3T3 conditions (red circles). MEFs cultured under the Std-3T3 conditions (black circles) as in Figure 1a were superimposed for comparison of the time needed to acquire immortality. Representative images of MEFs during the process of acquiring immortality are also shown. **B,C.** Tetraploidy development in immortalized MEFs (IP2) was observed by flow-cytometry (**B**) and Giemsa staining (**C**). **D.** Growth acceleration-associated cell cycle progression and H2AX induction. To determine the effect of serum induction on H2AX expression and cell cycle progression, senescent MEFs at P8 were incubated in serum-free medium for 24 h and harvested after exposure to complete medium for various times. H2AX expression increased with increasing PCNA and histone H3, which suggests that the expression of these chromatin factors was associated with S phase entry. To detect H2AX levels in these MEFs at P8, the H2AX signal was visualized by longer exposure. **E.** DNA lesions characterized by γH2AX foci were induced in MEFs (red arrowheads) after exposure to complete medium as in **D**. **F,G.** H2AX status in immortalized MEFs was determined by Western blotting (**F**) and immunofluorescence (**G**), revealing H2AX recovery. **H.** DNA replication stress-associated H2AX diminution was compared between normal and immortalized MEFs as in Figure 2E, in which H2AX was not down-regulated after immortalization.

The above results illustrate that cellular quiescence is produced when cells maintain stable diploidy and diminished H2AX expression. In these cells, the H2AX level is less than 100-fold compared to that in actively growing cells. To study the effect of growth stimulation in cells with an H2AX-diminished quiescent status, complete medium (DMEM with 10% FBS) was added to quiescent MEFs prepared under tSD-3T3 conditions (Fig. 3A–C). In these cells, cell-cycle progression was initiated with the expression of PCNA and histones H3 and H2AX, which led to γH2AX foci formation (Fig. 3D, E). Abrogating quiescent status with complete medium resulted in the establishment of immortalized MEFs with tetraploidy (Fig. 3A–C). However, it took 30 days to initiate immortal passage in H2AX-diminished quiescent MEFs, while immortality was acquired in only 9 days for P9 MEFs under Std-3T3 conditions, suggesting that the H2AX-diminished quiescent status protected cells from immortalization. Supporting this argument, primary MEFs transfected with an H2AX expression vector also acquired immortality at an accelerated rate (Fig. S5A–C). Such H2AX-overexpression may induce the effect of DNA replication stress because immortality in H2AX-overexpressing MEFs were again developed with tetraploidy (Fig. S5D,E). Unexpectedly, H2AX status was totally recovered in actively growing, immortalized MEFs (Fig. 3F, G), which illustrates the association of H2AX status with growth activity. However, this also poses the question of how the down-regulation of H2AX expression in quiescent MEFs is reversed after immortalization.

### Immortalized cells no longer achieve H2AX diminution-associated quiescent status

To explore the effects of the change in H2AX status, the response of H2AX to DNA replication stress was compared between primary and immortalized MEFs. While H2AX in primary MEFs was down-regulated after HU treatment, this did not occur in immortalized MEFs (Fig. 3H), which indicates that H2AX diminution-associated quiescent cell status is not inducible after immortalization. Thus, quiescent status is preserved in cells with diminished H2AX expression and stable diploidy but is abrogated under continuous growth stimulation, inducing cell cycle progression and γH2AX foci formation, and eventually leading to immortality with tetraploidy and H2AX recovery. Since the Arf/p53 module is specifically mutated during MEF immortalization [22], p53 might be involved in H2AX down-regulation. In fact, unlike senescent normal cells, H2AX expression is relatively high (2–20% of total H2A) in cancer cells as well as in growing NHFs (10%) [28].

### H2AX diminution-associated quiescent status is produced by p53 and prohibits the development of immortality

**Figure 4:**
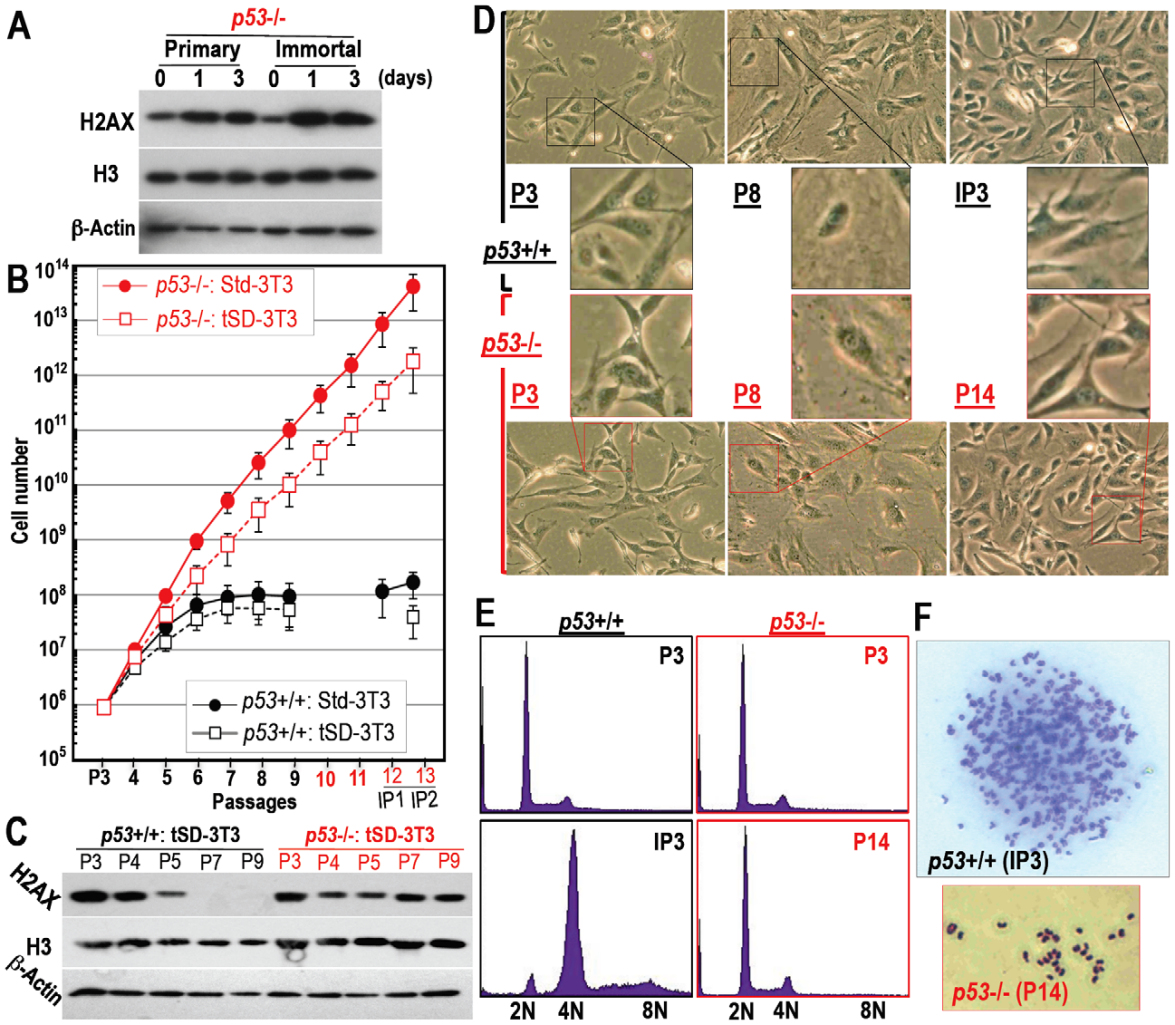
H2AX-diminished quiescent cell status is regulated by p53. **A.** DNA replication stress-associated H2AX diminution status was determined in *p53*-KO MEFs as in Figure 2E, in which H2AX was not down-regulated, even in primary MEFs. **B–F.** Primary *p53*-KO MEFs were cultured during the senescing and immortalizing processes (**B**). H2AX status was determined by Western blotting (**C**), morphological assessment (**D**), genomic status determined by flow-cytometry (**E**), and chromosome spread (**F**). Although *p53*-KO MEFs never showed major changes in H2AX expression, tetraploidization or growth arrest, *p53*-KO MEFs still exhibited a senescent morphology (P8) before achieving an immortalized morphology (P14).

To determine the involvement of p53 in H2AX down-regulation, *p53* knockout (KO) MEFs were cultured. Unlike normal primary MEFs, but similar to immortalized MEFs (Fig. 3H), H2AX expression in primary *p53*-KO-MEFs was not decreased by HU treatment (Fig. 4A). Furthermore, *p53*-KO-MEFs continuously grew, without change in H2AX status even under tSD-3T3 conditions (Fig. 4B, C). This indicates that H2AX in wild-type (WT)-MEFs is down-regulated by p53 to induce cellular quiescence and is recovered in immortalized MEFs in association with tetraploidization and mutation of the Arf/p53 module. Although *p53*-KO-MEFs did not undergo H2AX diminution-mediated growth arrest, these MEFs still exhibited a senescent morphology (Fig. 4D, see P8) and subsequently achieved an immortalized morphology (P14), which suggests the immortalization of *p53*-KO-MEFs via the senescent stage without growth arrest. This also indicates that a quiescent cell status is induced by p53 to protect cells from immortality.

### Mutation of the Arf/p53 module is induced with tetraploidization, triggered by DNA replication stress under moderately decreased H2AX levels in normal cells

Whereas *p53*-KO-MEFs are immortalized with diploidy (Fig. 4E, F), WT-MEFs are never immortalized only after tetraploidization [10] (Fig. 1B, C; Fig. 3B, C; Fig. 4E, F) and loss of Arf/p53 [22]. This suggests that the mutation of the Arf/p53 module in WT-MEFs is induced during tetraploidization. Supporting this argument, p53-dependent quiescence produced by diminished H2AX is maintained under diploidy preservation but abrogated after tetraploidization with mutation in the Arf/p53 module and the resulting H2AX recovery (Fig. 3). Therefore, normal WT-MEFs are protected from immortalization by a quiescent cell status, as long as the genome is preserved in diploidy. However, under continuous growth stimulation, tetraploidization also spontaneously arises in WT-MEFs but, unexpectedly, not in *p53*-KO-MEFs.

**Figure 5:**
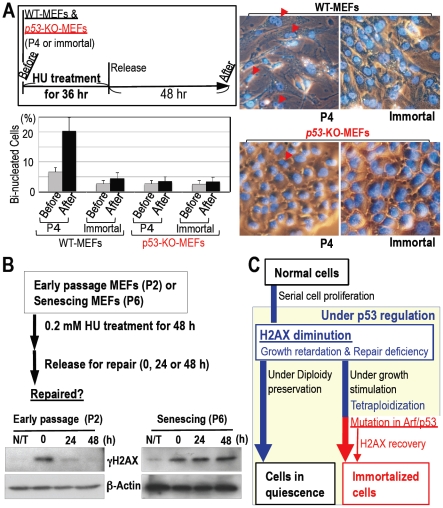
Increased risk of tetraploidization in normal MEFs. **A.** DNA replication stress-associated tetraploidization was determined in MEFs (P4) with the formation of bi-nucleated tetraploidy (red arrowhead) after 0.2 mM HU treatment as illustrated (top-left panel). Tetraploidization was efficiently induced during primary growth but not in immortalized MEFs or *p53*-KO-MEFs. **B.** Repair efficiencies of DNA replication stress-associated lesions were compared between early passage (P2) and senescing MEFs (P6) after 48 h hydroxyurea (HU) treatment. γH2AX signal was used as a marker of DNA lesions, in which γH2AX signal and β-Actin signals in senescing MEFs (P6) were detected only with over-exposure compared to early passage MEFs (P2), due to decreased H2AX levels during senescence. The reduction in γH2AX signal after release was only evident in early passage MEFs, which suggests that senescing cells are defective in resolving DNA replication stress. **C.** A model of MEFs under serial cell proliferation either undergoing quiescence or developing immortality. While MEFs that maintain quiescence and diploidy show diminished H2AX levels, MEFs developing immortality accumulate γH2AX foci.

As tetraploidization was observed at the senescent stage under conditions of continuous growth stimulation that induce DNA replication stress (Fig. 3), the underlying reason for tetraploidization in WT-MEFs but not in *p53*-KO-MEFs might be associated with the repair deficiency that also occurs in an H2AX-diminished background. To examine the tetraploidization risk under an H2AX-diminished background, MEFs of each type were treated with HU for 36 hours and the incidence of bi-nucleated tetraploidy formation was compared (Fig. 5A). As expected, HU treatment-associated H2AX diminution (Fig. 2E) resulted in tetraploidization in primary WT-MEFs but not in immortalized WT-MEFs or p53-KO-MEFs (Fig. 5A). Thus, although normal cells become quiescent with largely diminished H2AX under diploidy, senescing cells with residual H2AX under growth stimulating conditions are potentially at risk of developing tetraploidy in response to DNA replication stress.

Finally, to address changes in DNA replication stress-sensitivity during serial proliferation of normal MEFs, the repair efficiencies of DNA replication stress-associated lesions were compared between early passage and senescent MEFs with the decay of the γH2AX signal after release from HU treatment (Fig. 5B). Unlike early passage MEFs (P2), senescing MEFs (P6) were deficient in repairing HU-associated DNA lesions (Fig. 5B), in which MEFs show slow cell-cycle progression and residual H2AX expression. This is in contrast to quiescent MEFs with largely diminished H2AX level that show neither detectable cell cycle progression nor DNA replication stress. Thus, normal cells under serial proliferation decrease H2AX expression; thereby, cells slow growth activity and become defective in DNA repair. In such cells, cellular homeostasis is preserved by quiescence under largely diminished H2AX level regulated by p53 as long as diploidy is preserved. However, these cells are simultaneously at increased risk of tetraploidization with p53 dysfunction under continuous growth acceleration, resulting in the development of immortality and recovery of H2AX activity and cell growth (Fig. 5C).

## Discussion

The results of this study revealed the following novel concepts: (i) normal cells generally achieve quiescent status with diminished H2AX level both *in vitro* and *in vivo*, and this is regulated by p53; (ii) growth arrested normal cells with senescent morphology can be defined as either (a) those in a continuous quiescent status with largely diminished H2AX level or (b) those in a transient status with inducing genomic instability and the resulting onset of immortality, under which cells accumulate γH2AX foci; (iii) to protect cells from immortality, one of the critical roles of p53 is the induction of growth-arrest via the down-regulation of H2AX with cellular quiescence. Cells in H2AX diminution-associated quiescence are shown in the cause of mature and premature senescence, during which cells show senescent morphology (Fig. S1), probably because these cells are repair defective (Fig. 5B). However such repair deficiency is also associated with genomic instability development under accelerated growth stimulation, resulting in immortality acquisition with Arf/p53 module mutation and H2AX recovery.

Since growth-arrested cellular status with senescent morphology is directly induced by H2AX-knockdown (Fig. 2F), H2AX down-regulation is involved in a cause of quiescent cellular status. On the other hand, residual H2AX-expression in senescent cells is an associated effect for tetraploidization and immortalization: residual H2AX in senescent cells are only observed under accelerated growth stimulation (Figs. 1 and 3), under which cells are subjected to DNA replication stress and exhibit γH2AX, resulting in tetraploidization. Thus, even though cells are morphologically senescent with no growth in total cell number, cellular statuses could be either cells developing genomic instability under continuous growth acceleration (Std-3T3) or continuously quiescent cells under occasional arrest (tSD-3T3).

Unlike highly accumulated p53 that induces apoptosis, the Arf/p53 module under normal conditions functions for longevity by suppressing tumors in mice and giving protection from immortalization in MEFs [22]. Here, our results illustrated that such cellular status is produced with H2AX diminution-associated quiescence by protecting from immortalization under normal p53 regulation but is abrogated by Arf/p53 module mutation that is induced with tetraploidization under continuous growth stimulation, resulting in recovery of H2AX and growth activity. Unlike cells undergoing apoptosis, cells preserving quiescence under normal conditions do not accumulate p53 protein [10], which is probably associated with p53 function expression for quiescent status preservation but not for apoptosis induction. Intriguingly, such p53-dependent H2AX diminution was only observed after cells reach growth arrest both *in vivo* and *in vitro* but not growing cells in early passages and in organs from young mice (Fig. 2). In accordance with this, the expression of p53 targets Sidt2 and Phlda3, which are likely associated with tumor suppression [32], were elevated after cells become H2AX diminution-associated quiescent (P7) compared to cells in early passage (P3) (Fig. S6). However, similar to p53 protein, the increase in p53 transcript is also limited (Fig. S6). Thus, p53 function is expressed for apoptosis with accumulated p53, otherwise for H2AX-diminution associated quiescent status preservation under normal regulation without accumulating p53.

Except for tumors associated with specific chromosomal translocation, development of most cancers as well as *in vitro* cellular transformation is associated with genomic instability of either CIN or MIN [2], [3]. Importantly, tetraploidization, a major initial form of CIN under a mismatch repair proficient background is induced with oncogenic stress by accelerated S-phase entry [10], leading to immortality acquisition in MEFs with mutation in the Arf/p53 module. Here, our results showed that quiescence could be preserved with largely diminished H2AX and diploidy preservation under the regulation of p53. Although H2AX down-regulation is only observed under functional p53 regulation, it is still unclear how p53 down-regulates H2AX. Our results showed the reduction of total H2AX transcript during the senescing process (Fig. S4) and a damage-induced decrease of H2AX protein under functional p53 regulation (Fig. 2E; Fig. 4A, B). Although p53 role for H2AX down-regulation is unclear, the regulation might be indirect because (1) there is no p53-binding site on the *H2AX* promoter, (2) there is no signal of the *H2AX* gene with ChIP-on-CHIP analyses against p53 [33], (3) H2AX expression does not associate with the activation level of p53 as we observed no association between H2AX expression and p53 activation (Fig. S7).

Together, our results provide a rationale for the regulation of cellular homeostasis preservation. By prohibiting immortality development and preserving quiescent cell status, p53 induces an H2AX diminution-mediated quiescent status. However, this status is abrogated by continuous growth stimulation, which results in the induction of genomic instability with mutation of the Arf/p53 module, which leads into H2AX recovery, the restoration of growth activity, and immortality acquisition (Fig. 5C).

## Methods

### Ethics Statement

Mice were treated in accordance with the Japanese Laws and the Guidelines for Animal Experimentation of National Cancer Center. All experiments were approved by The Committee for Ethics in Animal Experimentation of National Cancer Center (approval ID numbers: A59-09 and T07-038).

### Cell culture and tissue samples

Cells were cultured as described previously [34]. Both wild-type and *p53*-KO MEFs were prepared from day 13.5 embryos of wild type and *p53* ^(+/−)^ mice [35] as previously described [34] and cultured under the standard 3T3 (Std-3T3) passage protocol [36] or with the following modifications: tSD-3T3. Senescing MEFs (P6 or P8) were maintained under tSD-3T3 conditions for the experiments shown in Figures 2, 3, 4, 5. NHFs (normal human umbilical cord fibroblasts; HUC-F2, RIKEN BRL Cell Bank) were cultured under Std-3T3 conditions. Resveratrol treatment of NHFs was performed as for MEFs. For the H2AX shRNA study, the reported sequence oligonucleotide [37], [38] was inserted into the pSuper.retro.puro vector (Oligoengine) and the shRNA virus was then prepared using 293T cells. The virus was infected into NHF cells and selected with puromycin. Mouse tissue samples were prepared from mice at the ages indicated (Sankyo Labo Service).

### DNA damage and induction of replication stress

DSB damage was induced by neocarzinostatin (Pola Pharma, Tokyo, Japan) treatment. For induction of DNA replication stress, MEFs were treated with hydroxyurea (HU).

### Antibodies, immunostaining and Western blotting

Antibodies against γH2AX (JBW301, Upstate Biotechnology) and H2AX (Bethyl) were used for immunostaining and Western blot analysis. Antibodies against β-actin (AC-74, Sigma), PCNA (Santa Cruz) and histone H3 (ab1791, Abcam) were used for Western blot analysis. Prior to immunostaining with primary and secondary antibodies, cells were fixed with 4% paraformaldehyde for 10 min and permeabilized with 0.1% Triton X-100/PBS for 10 min. Western blot analysis and confocal microscopy were performed as described previously [10].

### Transcription level analyses with RT-PCR

Total RNA was extracted from MEFs with the RNeasy system (Sigma). RNA (0.8 µg) was reverse-transcribed using a cDNA Archive kit (Applied Biosystems) and subjected to PCR. The following PCR primers were used: H2axf, 5′-TTGCTTCAGCTTGGTGCTTAG-3′; H2axr, AACTGGTATGAGGCCAGCAAC; β-actinf, CATCCAGGCTGTGCTGTCCCTGTATGC; and β-actinr, GATCTTCATGGTGCTAGGAGCCAGAGC; Trp53-F, CGGATAGTATTTCACCCTCAAGATCCG; Trp53-R, AGCCCTGCTGTCTCCAGACTC; Sidt2-F, CGGAAGGCTGGTTTCTGAGTTTCCG; Sidt2-R, CTGTAAACGCCAAGGACCAGAA; Phlda3-F, CGGTCCATCTACTTCACGCTAGTGACCG; Phlda3-R, TGGATGGCCTGTTGATTCTTGA; Gapdh-F, AACTTTGGCATTGTGGAAGG; Gapdh-R, ATGCAGGGATGATGTTCTGG. The amplified products by Ampli*Taq* Gold (Applied Biosystems) were separated on a 2% agarose gel and visualized with ethidium bromide. Otherwise, real-time PCR assay was carried out using Power SYBER green PCR Master kit (ABI).

### Chromosome spreads

Mitotic cells were prepared by treatment with 20 ng/ml nocodazole for 6 h and then collected. The collected cells were swollen hypotonically with 75 mM KCl for 15 min, and then fixed with Carnoy's solution (75% methanol/25% acetic acid) for 20 min. After changing the fixative once, the cells were dropped in Carnoy's solution onto glass slides and air-dried. The slides were stained with 4% Giemsa (Merck) solution for 10 min, washed briefly in tap water, and air-dried.

## Supporting Information

Figure S1 **Representative images of MEFs during the lifespan.** MEFs cultivated as in Figure 1A top lead into either immortality development under Std-3T3 or quiescence preservation under tSD-3T3. After serial cultivation, MEFs become morphologically senescent, i.e., flattened and enlarged morphology (P9) under both Std-3T3 and tSD-3T3 conditions. While continuous MEF-culture under tSD-3T3 preserved the quiescent status with continuously senescent morphology, continuous MEF-culture under Std-3T3 lead to the sporadic emergence of immortalized colony from the senescent MEFs. Immortalized MEFs (IP2) are morphologically escaped from senescence and rather similar to that in early passage (P3).
(TIF)

Figure S2 **H2AX diminution is also observed in adult mice organs.** Samples were prepared from five week (5W), five month (5M) and seven- or nine-month-old mice (7M or 9M). Compared to five months old organs, H2AX protein level is diminished in Testis (9M), Brain (7M), and Colon (7M), in which the diminution levels are lower than those in Liver, Spleen, and Pancreas. In Heart and Thymus, H2AX levels did not altered the alteration in through 5 weeks old to 7 or 9 months old.
(TIF)

Figure S3 **H2AX diminution is also shown in damage induced premature senescence.** Premature senescence was induced with NCS treatment as shown schematically in the top, in which each red arrowhead represents 100 ng/µl NCS treatment. Premature senescence by damage was induced with H2AX diminution, in which cells showed typical senescent morphology of flattened and enlarged.
(TIF)

Figure S4 **H2AX transcript is decreased in quiescent MEFs.** Decrease in H2AX mRNA level in senescing MEFs was observed by RT-PCR (right panel) and is compared with protein diminution (left panel).
(TIF)

Figure S5 **H2AX over-expression accelerates immortality development in MEFs with tetraploidy.** **A.** Experimental scheme of H2AX over expression. After transfection of H2AX-over expressing (H2AX-OE) or empty control vectors into early passage MEFs (P3), the transformed MEFs were selected, re-plated, and maintained in complete medium until immortalized cells appeared. **B.** Growth curves of MEFs during the experiments in A. MEFs before transfection and re-plating, MEFs transfected with H2AX-over-expressing vector, and MEFs transfected with empty control vector are indicated by black closed squares, red open circles, and black open diamonds, respectively. MEFs over-expressing H2AX showed accelerated development of immortality. **C.** H2AX status was determined as indicated in the figure. Although senescence was induced in the transfected and selected MEFs, H2AX over-expressing MEFs show higher levels of H2AX after the selection resulting in the development of immortality with H2AX recovery. **D.** Representative MEF images during accelerated immortality development with H2AX over-expression and controls. MEFs transfected with the H2AX over-expressing vector showed an efficient escape from senescence, while MEFs carrying the negative control vectors remained senescent with a flattened and enlarged morphology. **E,F.** Genomic instability status in immortalized MEFs (IP3) that were developed with H2AX over-expression was assessed by flow-cytometry (**E**) and Giemsa staining of M-phase chromosome (**F**).
(TIF)

Figure S6 **p53 expression in senescing MEFs.** To determine p53 expression in the cause of senescence, the expression levels of p53 and the targets (Sidt2 and Phlda3) that are likely associated with tumor suppression were compared between early passage (P2) and senescent MEFs (P7) under tSD-3T3 conditions. Along with H2AX diminution under p53 proficient background after serial cultivation, the expressions of Sidt2 and Phlda3 were observed in senescent MEFs (P7), in which the change in the expressed p53 transcript is limited.
(TIF)

Figure S7 **p53 activation shown by miR34a expression in primary wt-MEFs after damage is not directly associated with H2AX expression levels at least for transcript regulation.** **A.** To confirm p53 dependent DNA damage response, wt- and p53^−/−^-MEFs in primary and immortal were treated with 200 ng/ml neocarzinostatin (NCS) for 6 hours and the expression of p53-target miR34a was assessed. As expected, miR34a expression was shown after NCS treatment in primary wt-MEFs (wild type) but neither in immortalized wt-MEFs nor in p53^−/−^-MEFs. **B.** To determine the p53-activation associated change in the expression levels of H2AX transcript, mRNA levels of H2AX in MEFs treated as in **A** were analyzed. Whereas p53 is activated after NCS treatment in primary wt-MEFs, H2AX transcript levels were stable, suggesting no direct regulation by p53 transcription factor for H2AX expression. The PCR primers for miR34a were used from miRNA-specific primers (ABI) with snoRNA202 (ABI) for the control. Real-time PCR assay was carried out TaqMan microRNA assay kit (ABI).
(TIF)
