# Supplementary figures and images for: Onset of Quiescence Following p53 Mediated Down-Regulation of H2AX in Normal Cells
Source: PLoS One. 2011 Aug 12;6(8):e23432. doi: 10.1371/journal.pone.0023432 (PMC3155552; doi:10.1371/journal.pone.0023432)

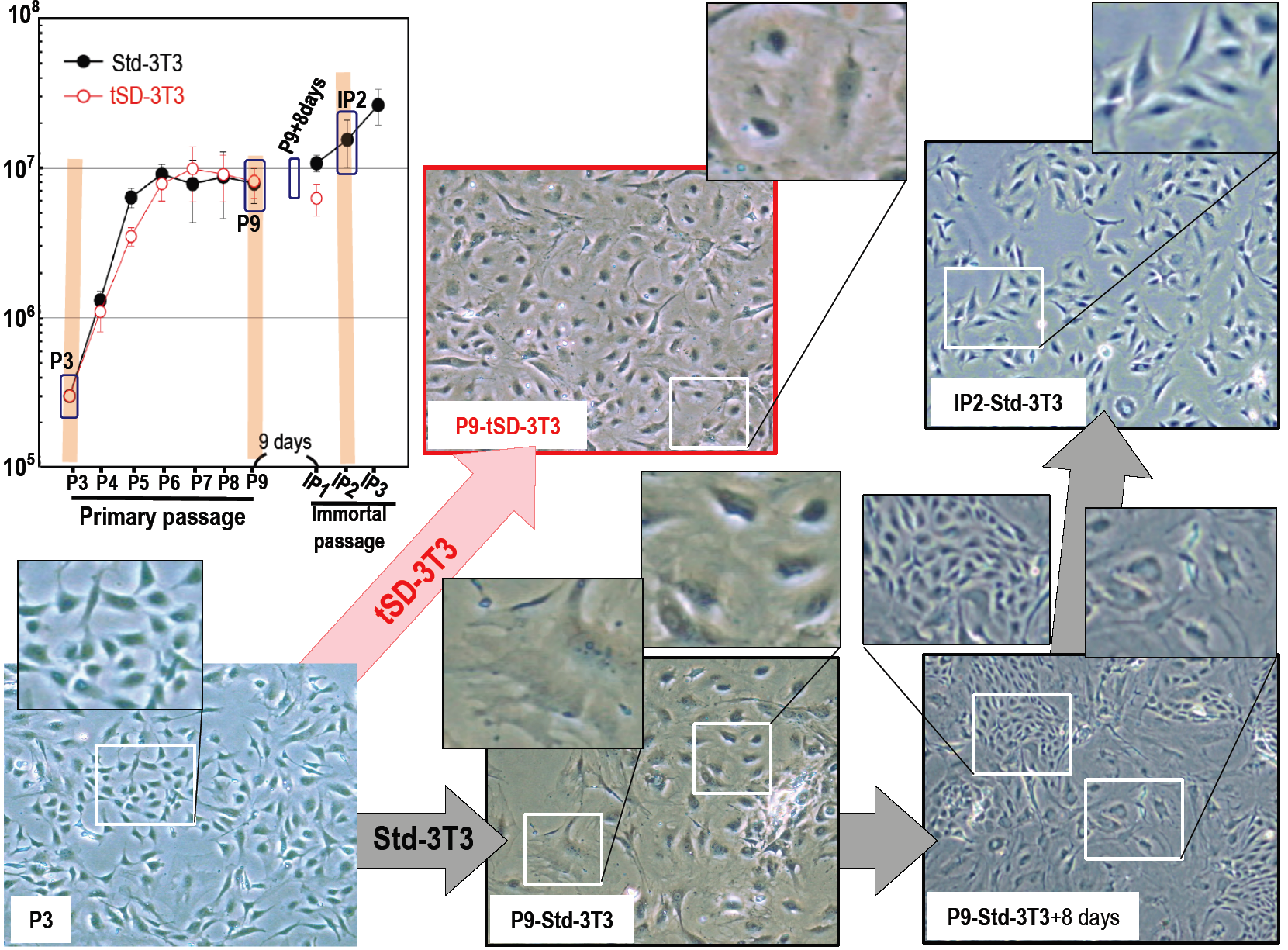

Supplement: Figure S1 — Representative images of MEFs during the lifespan. MEFs cultivated as in Figure 1A top lead into either immortality development under Std-3T3 or quiescence preservation under tSD-3T3. After serial cultivation, MEFs become morphologically senescent, i.e., flattened and enlarged morphology (P9) under both Std-3T3 and tSD-3T3 conditions. While continuous MEF-culture under tSD-3T3 preserved the quiescent status with continuously senescent morphology, continuous MEF-culture under Std-3T3 lead to the sporadic emergence of immortalized colony from the senescent MEFs. Immortalized MEFs (IP2) are morphologically escaped from senescence and rather similar to that in early passage (P3). (TIF) [file pone.0023432.s001.tif]

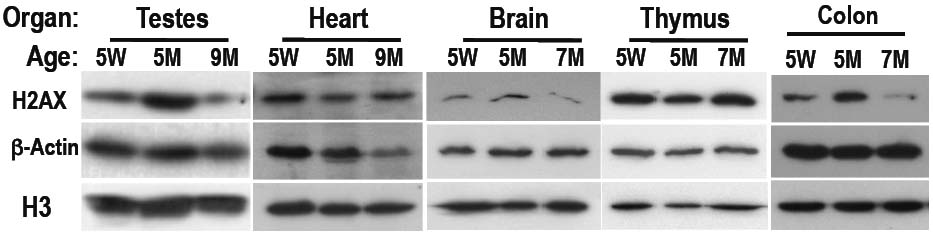

Supplement: Figure S2 — H2AX diminution is also observed in adult mice organs. Samples were prepared from five week (5W), five month (5M) and seven- or nine-month-old mice (7M or 9M). Compared to five months old organs, H2AX protein level is diminished in Testis (9M), Brain (7M), and Colon (7M), in which the diminution levels are lower than those in Liver, Spleen, and Pancreas. In Heart and Thymus, H2AX levels did not altered the alteration in through 5 weeks old to 7 or 9 months old. (TIF) [file pone.0023432.s002.tif]

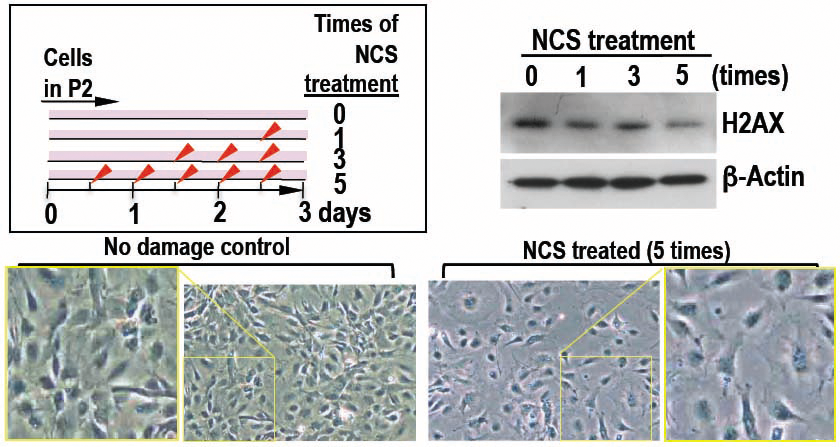

Supplement: Figure S3 — H2AX diminution is also shown in damage induced premature senescence. Premature senescence was induced with NCS treatment as shown schematically in the top, in which each red arrowhead represents 100 ng/µl NCS treatment. Premature senescence by damage was induced with H2AX diminution, in which cells showed typical senescent morphology of flattened and enlarged. (TIF) [file pone.0023432.s003.tif]

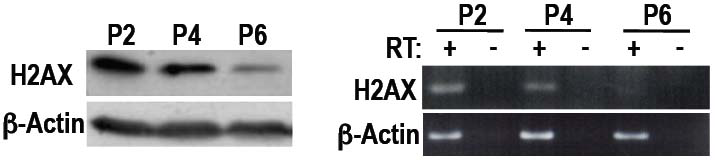

Supplement: Figure S4 — H2AX transcript is decreased in quiescent MEFs. Decrease in H2AX mRNA level in senescing MEFs was observed by RT-PCR (right panel) and is compared with protein diminution (left panel). (TIF) [file pone.0023432.s004.tif]

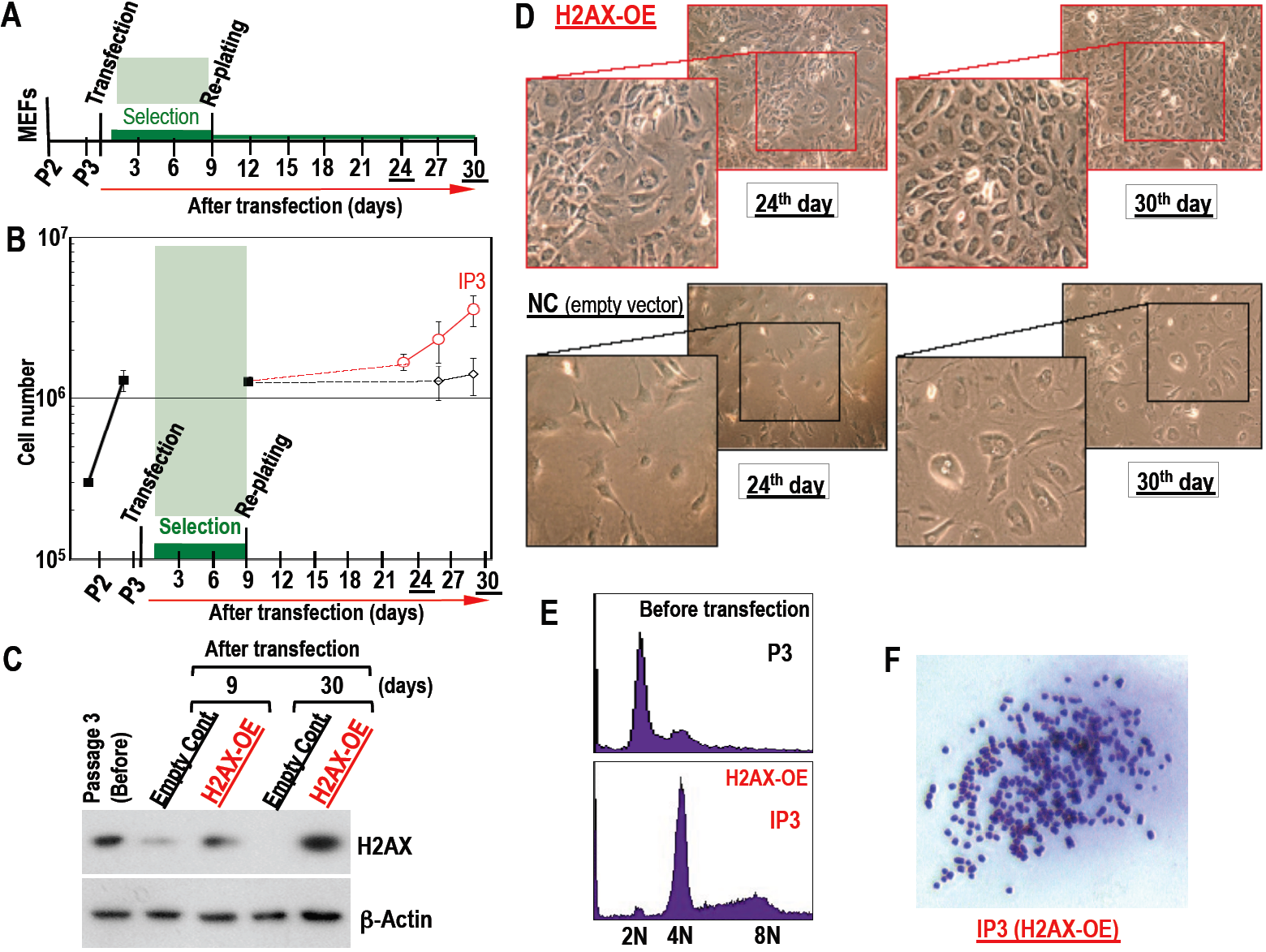

Supplement: Figure S5 — H2AX over-expression accelerates immortality development in MEFs with tetraploidy. A. Experimental scheme of H2AX over expression. After transfection of H2AX-over expressing (H2AX-OE) or empty control vectors into early passage MEFs (P3), the transformed MEFs were selected, re-plated, and maintained in complete medium until immortalized cells appeared. B. Growth curves of MEFs during the experiments in A. MEFs before transfection and re-plating, MEFs transfected with H2AX-over-expressing vector, and MEFs transfected with empty control vector are indicated by black closed squares, red open circles, and black open diamonds, respectively. MEFs over-expressing H2AX showed accelerated development of immortality. C. H2AX status was determined as indicated in the figure. Although senescence was induced in the transfected and selected MEFs, H2AX over-expressing MEFs show higher levels of H2AX after the selection resulting in the development of immortality with H2AX recovery. D. Representative MEF images during accelerated immortality development with H2AX over-expression and controls. MEFs transfected with the H2AX over-expressing vector showed an efficient escape from senescence, while MEFs carrying the negative control vectors remained senescent with a flattened and enlarged morphology. E,F. Genomic instability status in immortalized MEFs (IP3) that were developed with H2AX over-expression was assessed by flow-cytometry (E) and Giemsa staining of M-phase chromosome (F). (TIF) [file pone.0023432.s005.tif]

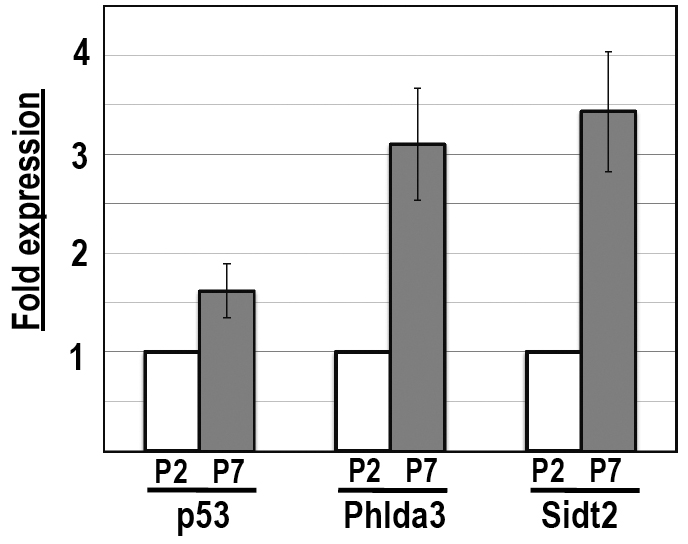

Supplement: Figure S6 — p53 expression in senescing MEFs. To determine p53 expression in the cause of senescence, the expression levels of p53 and the targets (Sidt2 and Phlda3) that are likely associated with tumor suppression were compared between early passage (P2) and senescent MEFs (P7) under tSD-3T3 conditions. Along with H2AX diminution under p53 proficient background after serial cultivation, the expressions of Sidt2 and Phlda3 were observed in senescent MEFs (P7), in which the change in the expressed p53 transcript is limited. (TIF) [file pone.0023432.s006.tif]

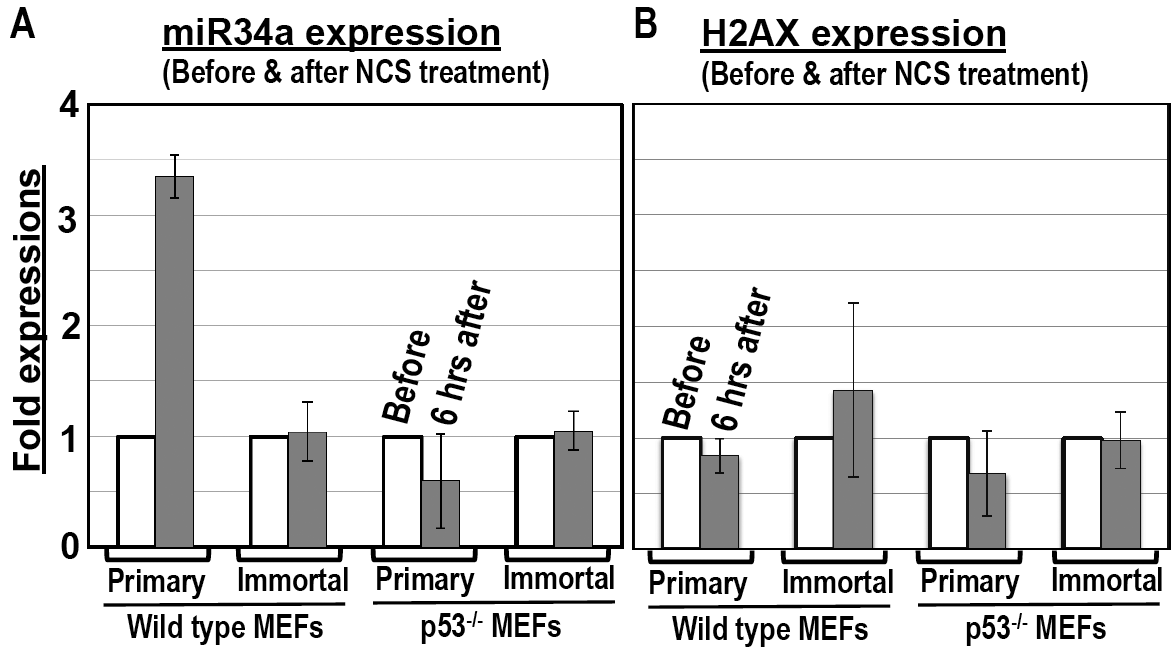

Supplement: Figure S7 — p53 activation shown by miR34a expression in primary wt-MEFs after damage is not directly associated with H2AX expression levels at least for transcript regulation. A. To confirm p53 dependent DNA damage response, wt- and p53−/−-MEFs in primary and immortal were treated with 200 ng/ml neocarzinostatin (NCS) for 6 hours and the expression of p53-target miR34a was assessed. As expected, miR34a expression was shown after NCS treatment in primary wt-MEFs (wild type) but neither in immortalized wt-MEFs nor in p53−/−-MEFs. B. To determine the p53-activation associated change in the expression levels of H2AX transcript, mRNA levels of H2AX in MEFs treated as in A were analyzed. Whereas p53 is activated after NCS treatment in primary wt-MEFs, H2AX transcript levels were stable, suggesting no direct regulation by p53 transcription factor for H2AX expression. The PCR primers for miR34a were used from miRNA-specific primers (ABI) with snoRNA202 (ABI) for the control. Real-time PCR assay was carried out TaqMan microRNA assay kit (ABI). (TIF) [file pone.0023432.s007.tif]
